# FMT and Bifidobacterium quadruple viable tablets supplementation modulate gut microbiota to ameliorate high-altitude exposure induced colonic injury

**DOI:** 10.3389/fmicb.2026.1856635

**Published:** 2026-09-10

**Authors:** Yuemei Sun, Fuyixuan Zheng, Qinqin Long, Junfei Cheng, Qian Ji, Rong Wang, Wenbin Li

**Affiliations:** 1 School of Pharmacy, Lanzhou University, Lanzhou, China; 2 Key Laboratory of the Plateau Medicial, The 940 Hospital of Joint Logistics Support, PLA, Lanzhou, China

**Keywords:** Bifidobacterium quadruple viable tablets (BQT), colonic inflammation, fecal microbiota transplantation (FMT), gut microbiota, high-altitude hypoxia, intestinal barrier

## Abstract

High-altitude hypoxic environments can induce significant gut microbiota dysbiosis, which in turn triggers colonic inflammation and intestinal barrier injury. This study aims to elucidate the core driving role of the gut microbiota in high-altitude induced colonic injury and to verify the protective effects of Bifidobacterium quadruple viable tablets (BQT). Here, we exposed C57BL/6 mice to acute and subacute exposure in high-altitude environment. High-altitude hypoxia led to colonic tissue damage, including epithelial cell shedding and inflammatory cell infiltration, and significantly reduced gut microbiota diversity in mice, manifested as a decreased Firmicutes/Bacteroidota ratio and increased abundances of Proteobacteria and Verrucomicrobiota. Fecal microbiota transplantation (FMT) improved colonic tissue morphology, reduced inflammatory cytokine levels, and enhanced intestinal barrier function. BQT intervention effectively alleviated colonic inflammation and reversed the down-regulation of tight junction proteins (ZO-1, Occludin, and Claudin-1) induced by hypoxia. In conclusion, gut microbiota dysbiosis is a key factor in high-altitude induced colonic inflammatory injury. FMT and BQT ameliorate high-altitude induced intestinal damage by restoring gut microbiota homeostasis and repairing the intestinal barrier. These findings suggest that supplementation with BQT may serve as a candidate microecological strategy for the prevention and treatment of intestinal injury in high-altitude populations.

## 1 Introduction

The plateau is characterized by a unique ecological environment, with hypoxia, low atmospheric pressure, and high radiation as its primary features. Among these, hypoxia is the core environmental factors, which can impair intestinal barrier integrity and induce gut microbiota dysbiosis (Han et al., 2022; Karl et al., 2018). These alterations facilitate the translocation of bacterial antigens such as lipopolysaccharide (LPS) from the intestinal lumen into the systemic circulation, striggering gastrointestinal symptoms associated with acute mountain sickness (AMS). In severe cases, this may progress to systemic inflammatory response syndrome or even multiple organ failure (Luks et al., 2017; Qi et al., 2025). Epidemiological data indicate that 81.4% of high-altitude climbers and tourists experience gastrointestinal discomfort, including nausea and vomiting, posing a significant threat to the physical and mental health of individuals rapidly ascending to high altitudes (Chen et al., 2024). With the development of high-altitude tourism, engineering, and military activities, the incidence of gastrointestinal diseases has risen correspondingly. This trend underscores the urgent need to protect the health and work capacity of high-altitude populations. Therefore, investigating the pathogenic mechanisms underlying high-altitude intestinal injury and developing effective preventive and therapeutic strategies are of substantial practical importance.

High-altitude exposure markedly reduces visceral perfusion and blood oxygen levels, leading to hypoxia and oxidative stress (Pena et al., 2022). Intestinal barrier injury induced by these factors can explain a range of gastrointestinal symptoms that occur at high altitudes (Cheng et al., 2022; Li et al., 2024). The intestinal barrier serves as a critical defense line. Intestinal epithelial cells, reinforced by tight junction proteins, effectively prevent the entry of harmful substances into the internal environment. Tight junctions are multi-protein complexes formed through the interaction of various proteins and represent a key structural element in maintaining mucosal epithelial barrier integrity and permeability. These complexes primarily consist of transmembrane proteins, including occludin and claudins, as well as scaffold proteins of the zona occludens (ZO) family. ZO proteins facilitate the direct binding of occludin and claudins, thereby providing structural support for tight junction stability (Horowitz et al., 2023; Kuo et al., 2022; Slifer and Blikslager, 2020). The dynamic regulation of tight junctions is essential not only for maintaining barrier integrity but also for ensuring the normal absorption of nutrients and macromolecules. Our previous studies have confirmed that high-altitude environments can induce inflammatory damage to the colonic barrier, significantly increasing the risk of bacterial and toxin invasion *via* the gut, which further exacerbates tissue injury (Cheng et al., 2022).

As a vital component of the colonic biological barrier, the gut microbiota colonizes the colon and provides energy to colonic epithelial cells, playing a key protective role in maintaining colonic health (Gao et al., 2023; Ottria et al., 2026; Wang et al., 2024). Environmental changes can readily induce gut microbiota dysbiosis, and increased intestinal permeability may lead to the leakage of luminal contents, such as gram-negative bacteria carrying LPS. Upon interaction with local innate immune cells, including macrophages and monocytes, these microbial components can initiate inflammatory cascades, promoting the production and release of pro-inflammatory cytokines and thereby exacerbating intestinal damage (Hazime et al., 2025; Morris et al., 2014; Sun et al., 2021). Accumulating evidence suggests that hypoxic environment can significantly alter the composition and diversity of the gut microbiota. More than 80% of the gut microbiota typically belong to the phyla Firmicutes and Bacteroidota (Tingler and Engevik, 2025). Ma et al. (2025) conducted a large-scale longitudinal cohort study involving 406 healthy adult males. At the genus level, the acute phase was characterized by a significant enrichment of opportunistic pathogens, including *Ruminococcus, Gemmiger*, and *Parabacteroides*, whereas beneficial bacteria, such as short chain fatty acid producing genera including *Faecalibacterium, Roseburia*, and *Bifidobacterium*, were significantly reduced. Another study using a hypobaric oxygen chamber simulating an altitude of 3,500–4,000 m reported that microbial richness in the colon was significantly reduced under hypobaric hypoxic condition, whereas microbial richness and diversity in the duodenum and jejunum remained largely unchanged. This finding suggests that the colon may be the primary intestinal segment affected by high-altitude environments (Zhao et al., 2024). This observation may also explain why individuals rapidly acclimatizing to high altitudes are prone to digestive symptoms such as nausea, vomiting, indigestion, and bloating, and further indicates that the gut microbiota may represent a key, modifiable target for mitigating high-altitude induced intestinal injury.

Probiotic supplementation is commonly used for the prevention and treatment of intestinal diseases (Campaniello et al., 2023; Kumar et al., 2025; Zeng et al., 2025). Bifidobacterium quadruple viable tablets (BQT) have been widely used to treat diseases associated with gut microbiota dysbiosis, and their safety and tolerability have been well established. BQT supplementation regulates gut microbiota homeostasis. However, its protective effects and specific mechanisms of action in the context of highaltitude hypoxia induced intestinal injury remain unexplored. Accordingly, the present study focuses on intestinal injury in high-altitude environments. By investigating the effects of high-altitude exposure on the gut microbiota in mice and employing pseudo-germ-free (PGF) models combined with fecal microbiota transplantation (FMT), we aim to elucidate the relationship between colonic injury and gut microbiota dysbiosis. Furthermore, we will administer BQT to evaluate their efficacy in restoring colonic barrier integrity and alleviating inflammatory damage. This study seeks to clarify the central role of the gut microbiota in driving high-altitude colonic inflammatory injury and to provide a theoretical foundation for the development of microbiological interventions targeting high-altitude intestinal damage through microecological regulation.

## 2 Materials and methods

### 2.1 Animal ethics

Eight-week-old male specific pathogen-free (SPF) C57BL/6J mice, weighing 20 ± 2 g, were purchased from Sibeifu (Beijing) Biotechnology Co., Ltd. [animal production license number: SCXK (Jing) 20240001]. All experimental procedures were reviewed and approved by the Ethics Committee for Laboratory Animals of the 940th Hospital of the Joint Logistics Support Force (ethics approval No.: 2023KYLL082). The welfare and rights of laboratory animals were ensured throughout the study.

During the experiment, the room temperature was maintained at 20–25 °C, relative humidity at 40%−60%, and a 12 h light/12 h dark cycle was applied, such that the housing environment conformed to SPF-grade standards. Mice were given *ad libitum* access to food and water. The control mice were housed in an SPF-grade animal facility in Lanzhou, Gansu Province, China. The high-altitude group mice were transported from Lanzhou (1,500 m) to the high-altitude SPF-grade field animal laboratory in Batang Township, Yushu Tibetan Autonomous Prefecture, Qinghai Province (4,010 m) using a thermostatically controlled box truck. The entire acute ascent process was completed within 12 h.

### 2.2 Animal grouping and experimental design

The animal experiments in this study mainly consisted of two parts: establishment of FMT model and BQT intervention. For the FMT model establishment, a total of 63 mice were randomly divided into 7 groups: control group (Con), control PGF model group (CP), high-altitude 12 h group (H12h), high-altitude 12 h PGF model group (H12P), high-altitude 8 d group (H8d), high-altitude 8 d PGF model group (HP), and high-altitude 8 d PGF model + FMT group (HP+FMT). For the BQT intervention, 45 mice were randomly assigned to 5 groups: control group, 12 h high-altitude group (H12h), 12 h high-altitude BQT group (H12Y), an 8 d high-altitude group (H8d), and an 8 d high-altitude BQT group (HY). All high-altitude groups were housed in a specific pathogen-free (SPF) animal laboratory at an altitude of 4,010 m in Yushu, Qinghai Province, China. The BQT preparation (Siliankang^®^, Hangzhou Yuanda Bio-Pharmaceutical Co., Ltd., National Drug Approval No. S20060010) was administered at a dose of 585 mg/kg once daily by intragastric gavage. The dose was converted from the recommended human clinical dosage based on body surface area, following pharmacological methodology.

### 2.3 Establishment of PGF model

Experimental mice were housed in a room disinfected by ultraviolet (UV) irradiation. Mice were administered a combination of four antibiotics by gavage twice daily for 9 days, with the following formulation: moxifloxacin (52 mg/kg), roxithromycin (39 mg/kg), metronidazole (100 mg/kg), and amoxicillin (195 mg/kg). During the modeling period, the feed and bedding were disinfected and changed once daily to strictly maintain a sterile environment. Immediately after modeling, fresh fecal samples were collected from each group of mice. The fecal samples were subjected to smear preparation and examined under a microscope for bacterial quantity and morphology to verify the successful establishment of the PGF model.

### 2.4 Establishment of FMT model

Following the fecal microbiota transplantation method used in other studies (Shang et al., 2022), healthy mouse feces were collected. A 200 mg aliquot was dissolved in 5 ml of 0.1 M PBS (containing 10% sterile glycerol), vortexed, and centrifuged at 1,000×g for 10 min at 4 °C. The supernatant was aliquoted and stored at −80 °C until use. Each mouse received 0.2 ml by gavage twice daily for 8 days. Fecal samples were then collected for Gram staining to preliminarily assess bacterial colonization. After confirming that the staining results were similar to those of the control group, successful model establishment was further confirmed by 16S rRNA analysis.

### 2.5 Collection and processing of colon tissue and fecal samples

Prior to dissection, fecal samples were collected from each group of mice into enzyme-free tubes. For histological analysis, two mice from each group were euthanized by cervical dislocation, and colon tissue segments of approximately 2 cm in length were collected and fixed in 4% paraformaldehyde for subsequent HE staining. The remaining mice were euthanized by cervical dislocation, and their colon tissues were collected into EP tubes. All samples were snap-frozen in liquid nitrogen, transported on dry ice, and subsequently stored at −80 °C upon arrival.

### 2.6 16S rRNA analysis of mouse feces

Fecal samples (0.25–0.5 g) were collected from mice in the control group (Con), the high-altitude 12 h group (H12h), the high-altitude 8 d group (H8d), and the high-altitude PGF model with FMT 8 d group (HP + FMT). Samples were placed into 2 mL centrifuge tubes. A commercial DNA extraction kit (Servicebio, China) was used according to the manufacturer's instructions. Briefly, 500 μL Buffer A, 5 μL RNase A, and 60 μL Buffer B were added to each tube. The samples were homogenized using a high-speed grinder, then incubated at 70 °C for 15 min to achieve lysis. After vortexing, the samples were incubated at 70 °C for an additional 15 min. Subsequent 16S rRNA gene sequencing and bioinformatics analysis were performed by Servicebio.

### 2.7 HE staining of colon tissue

Colon tissue segments (2 cm in length) were collected from mice and fixed in 4% paraformaldehyde. The tissues were then dehydrated through a graded ethanol series, embedded in paraffin, and sectioned at a thickness of 4 μm for hematoxylin and eosin (HE) staining. Finally, the stained sections were examined under a light microscope, and images were acquired.

### 2.8 Determination of inflammatory factors in colon tissue

Colon tissue samples were retrieved from a −80 °C freezer. Each sample (30–35 mg) was weighed and mixed with phosphate-buffered saline (PBS) at a ratio of 1:9 (w/v, tissue to PBS). The mixture was thoroughly homogenized using a high-speed grinder and then centrifuged at 5,000×g for 10 min. The supernatant was collected, and the levels of IFN-γ, IL-6, TNF-α, IL-10, IL-12, and IL-1β were measured using corresponding ELISA kits according to the manufacturer's instructions.

### 2.9 Real-time PCR analysis of gene expression

Approximately 50 mg of frozen colon tissue was collected, and total RNA was extracted using Trizol reagent following the manufacturer's instructions. The purity and concentration of the isolated RNA were determined with a UV spectrophotometer. Subsequently, 1 μg of total RNA was reverse transcribed into complementary DNA (cDNA) using a reverse transcription kit in strict accordance with the manufacturer's protocols, and the resulting cDNA products were stored at −20 °C for subsequent experimental analysis. Primers specific for the target genes Claudin-1, Occludin, and ZO1, as well as the internal reference gene β-actin, were designed and synthesized by Sangon Biotech Co., Ltd. (Shanghai, China). The primer sequences were as follows: for Claudin-1, forward 5′-TGCCCCAGTGGAAGATTTACT-3′ and reverse 5′-CTTTGCGAAACGCAGGACAT-3′; for Occludin, forward 5′-TGAAAGTCCACCTCCTTACAGA-3′ and reverse 5′CCGGATAAAAAGAGTACGCTGG-3′; for ZO-1, forward 5′-GCTTTAGCGAACAGAAGGAGC-3′ and reverse 5′-TTCATTTTTCCGAGACTTCACCA-3′; and for β-actin, forward 5′- GTGACGTT-GACATCCGTAAAGA-3′ and reverse 5′GCCGGACTCATCGTACTCC-3′. Real-time PCR was conducted using the SYBR Green fluorescence method. The 20 μL reaction system consisted of 10 μL of TB Green Premix Ex Taq II, 0.4 μL of forward primer (10 μM), 0.4 μL of reverse primer (10 μM), 2 μL of cDNA template, and double-distilled water (ddH_2_O) to make up the final volume. The thermal cycling parameters were set as follows: initial denaturation at 95 °C for 30 s, followed by 40 cycles of denaturation at 95 °C for 5 s and annealing/extension at 60 °C for 30 s. A melting curve analysis was performed after the PCR to confirm the specificity of amplification. Using β-actin as the internal reference gene, the relative expression levels of the target genes were calculated using the 2^−^ΔΔCt method.

### 2.10 Western blot analysis of protein expression

Approximately 50 mg of frozen colon tissue was collected and mixed with 500 μL of RIPA lysis buffer containing protease inhibitors. After homogenization, the sample was centrifuged at 12,000×g for 10 min at 4 °C, and the supernatant was collected. The protein concentration was determined using a BCA assay to adjust the dilution ratio. The supernatant was then mixed with 4×loading buffer at a ratio of 3:1 (v/v), and the mixture was heated to denature the proteins. The denatured protein samples were aliquoted and stored at −80 °C for further use. Protein samples were separated by sodium dodecyl sulfate-polyacrylamide gel electrophoresis (SDS-PAGE) and then transferred onto 0.22 μm polyvinylidene fluoride (PVDF) membranes. The membranes were blocked with 5% freshly prepared non-fat milk at room temperature for 2 h to block nonspecific binding sites. After blocking, the membranes were incubated overnight at 4 °C with the corresponding primary antibodies. The primary antibodies used in this experiment were as follows: Claudin-1 (1:1000 dilution, Abcam), Occludin (1:1000 dilution, Abcam), ZO-1 (1:1000 dilution, Abcam), and β-actin (1:5000 dilution, Proteintech). Following incubation with primary antibodies, the membranes were washed three times with 1×TBST buffer (each wash lasting 5–10 min). Then, the membranes were incubated with HRP-conjugated secondary antibodies at room temperature for 2 h. Finally, the protein bands were visualized and quantified using Bio-Rad imaging system, and densitometric analysis was performed to calculate the relative expression level of each target protein normalized to β-actin.

### 2.11 Statistical analysis

Statistical analysis was performed using SPSS (version 21.0). Data are presented as mean ± SD. Comparisons among multiple groups were conducted using one-way analysis of variance (ANOVA). *P* < 0.05 was considered statistically significant. We first assessed the distribution of all quantitative data using normality tests and evaluated the homogeneity of variances using homogeneity of variance tests, to verify the assumptions required for ANOVA. For differential taxa screening in the microbiome data, FDR correction was uniformly applied.

## 3 Results

### 3.1 Effects of different durations of high-altitude exposure on the fecal gut microbiota in mice

**Figure 1:**
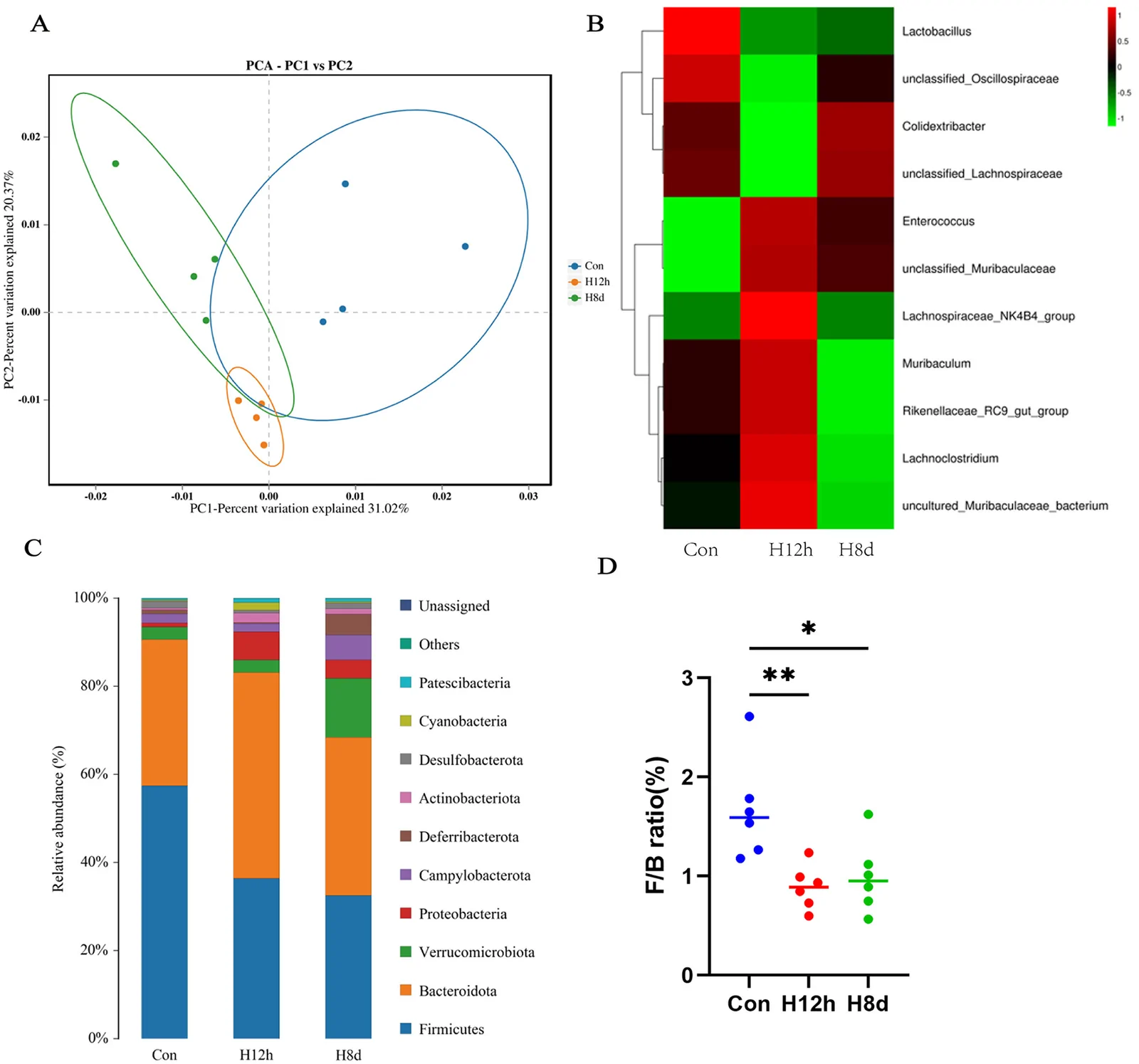
Effects of high-altitude exposure on fecal gut microbiota in mice. **(A)** PCA showed distinct separation among groups. **(B)** Heatmap of gut microbiota composition. **(C)** Relative abundance of microbiota. **(D)** The ratio of Firmicutes to Bacteroidota.

In this study, a mouse model of high-altitude intestinal microbiota dysbiosis was successfully established at an altitude of 4,010 m. Principal Component Analysis (PCA) was employed to comprehensively analyze the structure of the mouse gut microbiota. As shown in Figure 1A, the blue, orange, and green colors represent the control group, the high-altitude 12 h group, and the high-altitude 8 d group, respectively. The PCA results demonstrated a high degree of clustering among samples from the same environmental condition, indicating good reproducibility within groups. The spatial distribution of the control group was distinctly separated from the high-altitude groups, suggesting that high-altitude exposure induced significant alterations in the composition of the gut microbiota. Further analysis of microbial composition revealed that high-altitude exposure markedly affected the structure and relative abundance of the gut microbiota. At the genus level, as illustrated in Figure 1B, compared with the control group, the relative abundances of *Enterococcus* was increased in both the high-altitude 12 h and 8 d groups, whereas those of *Lactobacillus* was significantly decreased. Concurrently, at the phylum level (Figure 1C), the relative abundance of Firmicutes was significantly reduced, while Bacteroidota and Verrucomicrobiota were significantly elevated, consistent with the trends observed at the genus level. High-altitude exposure significantly reduced the Firmicutes to Bacteroidota ratio (F/B ratio), a key indicator of gut microbiota homeostasis (Figure 1D). The heatmap and species relative abundance analyses collectively indicate that high-altitude exposure can significantly alter the compositional structure and relative abundances of gut microbiota. A decreased F/B ratio has been associated with intestinal barrier dysfunction and enhanced inflammatory responses.

### 3.2 FMT alleviates colonic inflammatory injury by modulating gut microbiota in a high altitude environment

**Figure 2:**
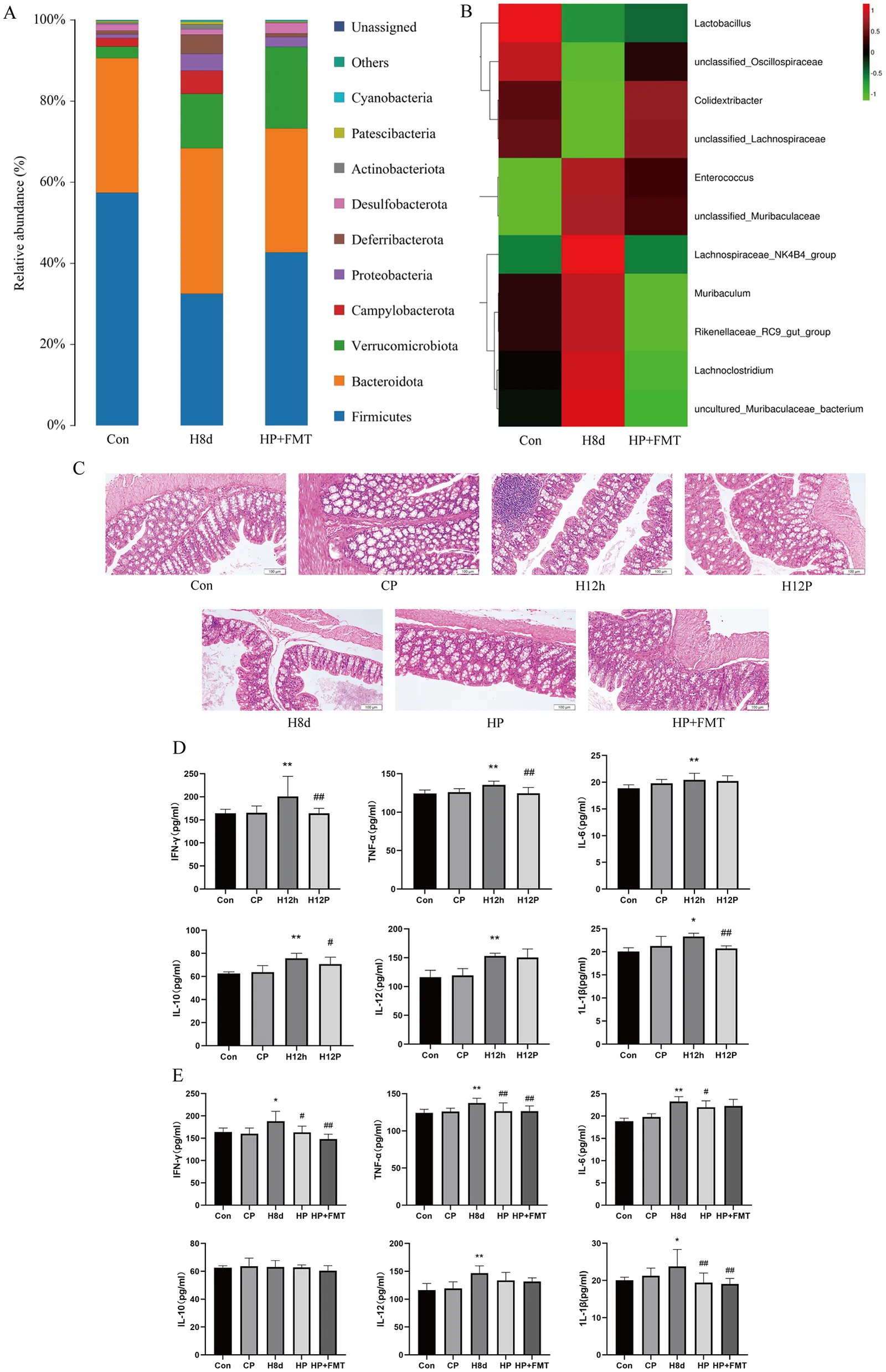
FMT modulates gut microbiota composition and colonic inflammatory responses in high-altitude exposed mice. **(A)** Relative abundance of gut microbiota after FMT. **(B)** Heatmap of gut microbiota composition after FMT. **(C)** HE staining of colon tissues from each experimental group (200×). **(D)** Expression of inflammatory factors in colon tissues of each experimental group after high-altitude exposure 12 h. Data were presented as mean ± SD (*n* = 7). ^*^Con vs. H12h, ^*^P < 0.05, ^**^P < 0.01. ^#^H12h vs. H12P, ^#^*P* < 0.05, ^*##*^*P* < 0.01. **(E)** Expression of inflammatory factors in colon tissues of each experimental group after high-altitude exposure 8 d. Data were presented as mean ± SD (*n* = 7). ^*^Con vs. H8d, ^*^P < 0.05, ^**^P < 0.01. ^#^H8d vs. HP, HP + FMT, ^#^*P* < 0.05, ^*##*^*P* < 0.01.

Our previous study demonstrated that exposure to a high-altitude environment for different durations significantly induces colonic barrier injury. To determine whether the high-altitude induced colonic barrier inflammatory injury is associated with the disruption of gut microbiota homeostasis, we established PGF and FMT model in the present study. As shown in Figure 2A, the results revealed that high-altitude exposure altered the composition and relative abundance of fecal microbiota in mice, characterized by an increase in Bacteroidota and a decrease in Firmicutes. Following FMT, the abundance of Bacteroidota was decreased, whereas that of Firmicutes was increased. Meanwhile, heatmap analysis indicated that FMT reversed the alterations in partial microbiota composition, verifying the successful establishment of the FMT model (Figure 2B). Interestingly, we observed a marked elevation in Verrucomicrobiota under high-altitude conditions. The primary genus of Verrucomicrobiota, *Akkermansia*, is known to exert beneficial effects on intestinal barrier function, which may represent a compensatory strategy for the host to adapt to hypoxic stress.

We subsequently examined the morphological changes in colonic tissues of PGF and FMT mice under high-altitude conditions. As shown in Figure 2C, HE staining showed that colonic epithelial cells in the control group were neatly arranged with intact intestinal mucosal epithelium and tight cell junctions. After antibiotic treatment to establish the PGF model, the colonic morphology remained relatively intact, and mucosal thickness were comparable to those in the control group, suggesting that the PGF model did not cause obvious colonic tissue damage. In contrast, mice exposed to high altitude for 12 h and 8 d exhibited obvious lymphocyte infiltration, decreased mucosal thickness, and shortened mucosal epithelium height in colonic tissues, indicating pathological injury induced by high-altitude exposure. Although mild lymphocyte infiltration was still observed in PGF mice exposed to high altitude for 12 h and 8 d, mucosal thickness was partially improved compared with the high-altitude control group. Notably, mice in the high-altitude 8 d PGF+ FMT group showed no lymphocyte infiltration, were tightly arranged and tissue damage was significantly alleviated, indicating that transplantation of normal gut microbiota effectively ameliorated high-altitude induced colonic injury. These histological findings preliminarily suggest that gut microbiota dysbiosis serves as a trigger or key mediator of high-altitude induced colonic pathological damage.

Furthermore, we investigated the effects of high-altitude exposure on colonic inflammatory cytokine responses. The results showed that colonic inflammatory cytokine levels in the PGF model group were similar to those in the control group with no significant differences, suggesting that establishment of the PGF model did not significantly affect colonic inflammatory cytokine expression. However, the levels of colonic inflammatory cytokines were markedly upregulated in mice exposed to high altitude for 12 h and 8 d (Figures 2D, E). In the 12 h and 8 d high-altitude groups, the levels of pro-inflammatory cytokines (IFN-γ, IL-6, TNF-α, IL-12, and IL-1β) were significantly elevated, and the anti-inflammatory cytokine IL-10 was also markedly upregulated, suggesting that high-altitude exposure triggers colonic inflammation accompanied by activation of a compensatory anti-inflammatory response. In PGF mice exposed to high altitude for 12 h and 8 d, the aforementioned pro-inflammatory cytokines were significantly downregulated, while IL-10 showed a reduction, indicating that depletion of the disturbed gut microbiota attenuates the inflammatory stimulus without substantially compromising the anti-inflammatory capacity. In the high-altitude 8-d PGF+FMT group, pro-inflammatory cytokines were downregulated to levels comparable to those in the control group, accompanied by a mild reduction in IL-10, suggesting that restoration of a healthy microbiota effectively protects colonic tissue from dysbiosis-driven injury and alleviates inflammation. Collectively, the results from PGF and FMT experiments further confirm that gut microbiota plays a crucial role in high-altitude-induced colonic inflammatory injury in mice.

### 3.3 Improvement of tight junction protein and mRNA expression in colon tissue by FMT in mice under high-altitude exposure

**Figure 3:**
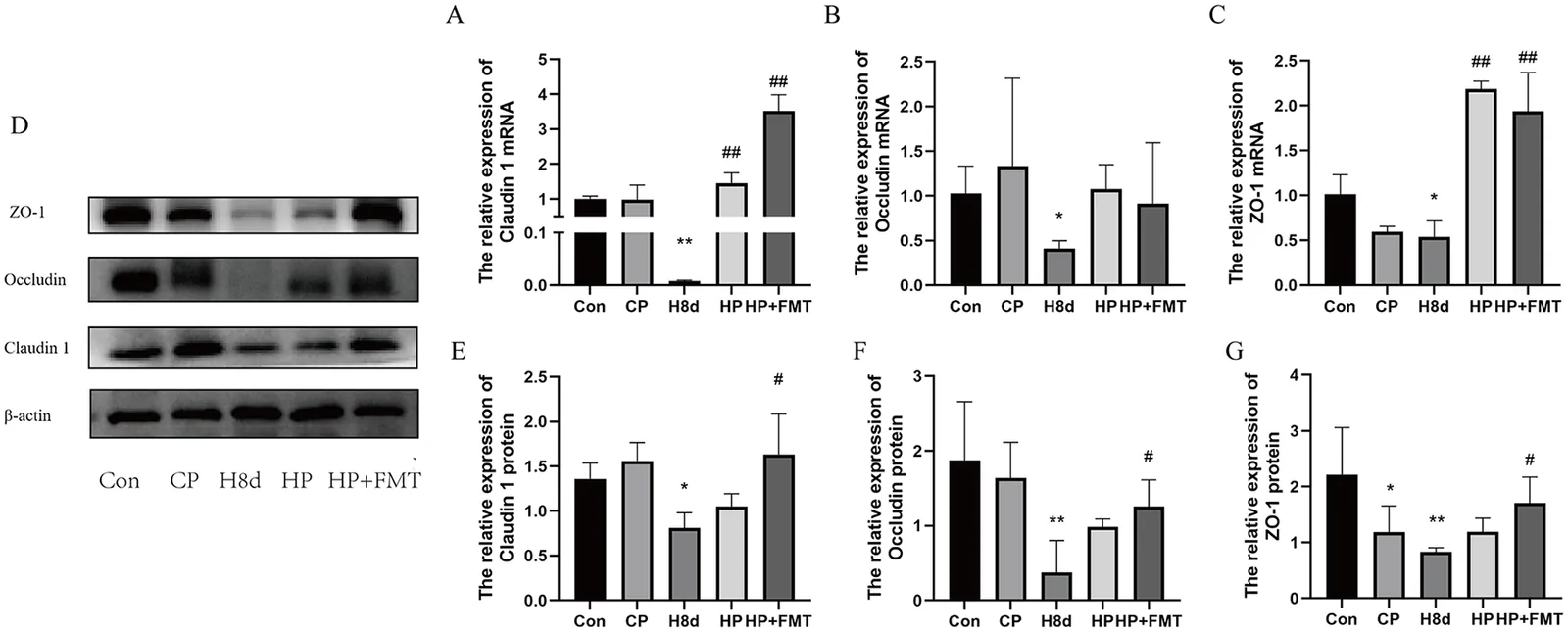
Expression of mRNA and corresponding protein levels of tight junction proteins in colon tissues of each group. **(A–C)** Relative mRNA expression levels of Claudin-1, Occludin, and ZO-1. **(D)** Representative Western blot bands of Claudin-1, Occludin, and ZO-1. **(E–G)** Relative protein expression levels of Claudin-1, Occludin, and ZO-1. ^*^Con vs. H8d, ^*^P < 0.05, ^**^P < 0.01. H8d vs. HP, HP+FMT, ^#^*P* < 0.05, ^*##*^*P* < 0.01.

As shown in Figures 3A–G), compared with the control group, Claudin 1, Occludin, and ZO-1 protein and mRNA expression levels of tight junction proteins in the colon tissue of mice in the control PGF (CP) group decreased to a certain extent, but the differences were not statistically significant (*P* > 0.05). This suggests that the establishment of the PGF model had no significant effect on the expression of colonic tight junction proteins in mice and would not interfere with the accuracy of subsequent experimental results. The protein and mRNA expression levels of Claudin 1, Occludin, and ZO-1 in the colon tissue of mice in the high-altitude 8d (H8d) group were significantly down-regulated (*P* < 0.05), with protein levels decreased by 40.51, 80.06, and 62.33%, respectively, and mRNA levels significantly decreased. These results indicate that high-altitude exposure can significantly inhibit the transcription and translation of colonic tight junction proteins in mice, leading to impaired colonic barrier function. Compared with the high-altitude group 8d (H8d), the protein and mRNA expression levels of the three tight junction proteins in the colon tissue of mice in the high-altitude 8 d PGF (HP) group were up-regulated to varying degrees. The protein levels of Claudin 1, Occludin, and ZO-1 were upregulated by 29.73, 163.06, and 42.84%, respectively, and mRNA levels were significantly upregulated (*P* < 0.05). The expression levels of tight junction proteins in the colon tissue of mice in the high-altitude 8 d PGF+FMT (HP+FMT) group were further increased. Compared with the high-altitude 8 d group, the protein levels of Claudin 1, Occludin, and ZO-1 were up-regulated by 101.91, 236.59, and 104.77%, respectively, and mRNA levels were significantly up-regulated (*P* < 0.05). Moreover, their expression levels were significantly higher than those in the high-altitude PGF (HP) group (*P* < 0.05). These findings indicate that after eliminating disturbed gut microbiota, transplantation of normal gut microbiota can more significantly promote the expression of tight junction proteins, exerting an important protective effect against high-altitude exposure induced colonic barrier injury in mice.

In conclusion, both elimination of disturbed gut microbiota and subsequent transplantation of normal gut microbiota can improve high-altitude exposure induced colonic barrier injury in mice, and the protective effect of fecal microbiota transplantation is more significant. From the perspective of regulating tight junction protein expression, gut microbiota plays a key regulatory role in the process of high-altitude expo-sure-induced colonic barrier dysfunction, providing an experimental basis for the subsequent exploration of the pathogenesis and intervention strategies of high-altitude colonic barrier injury.

### 3.4 BQT alleviates colonic barrier injury and inflammation under high-altitude conditions

**Figure 4:**
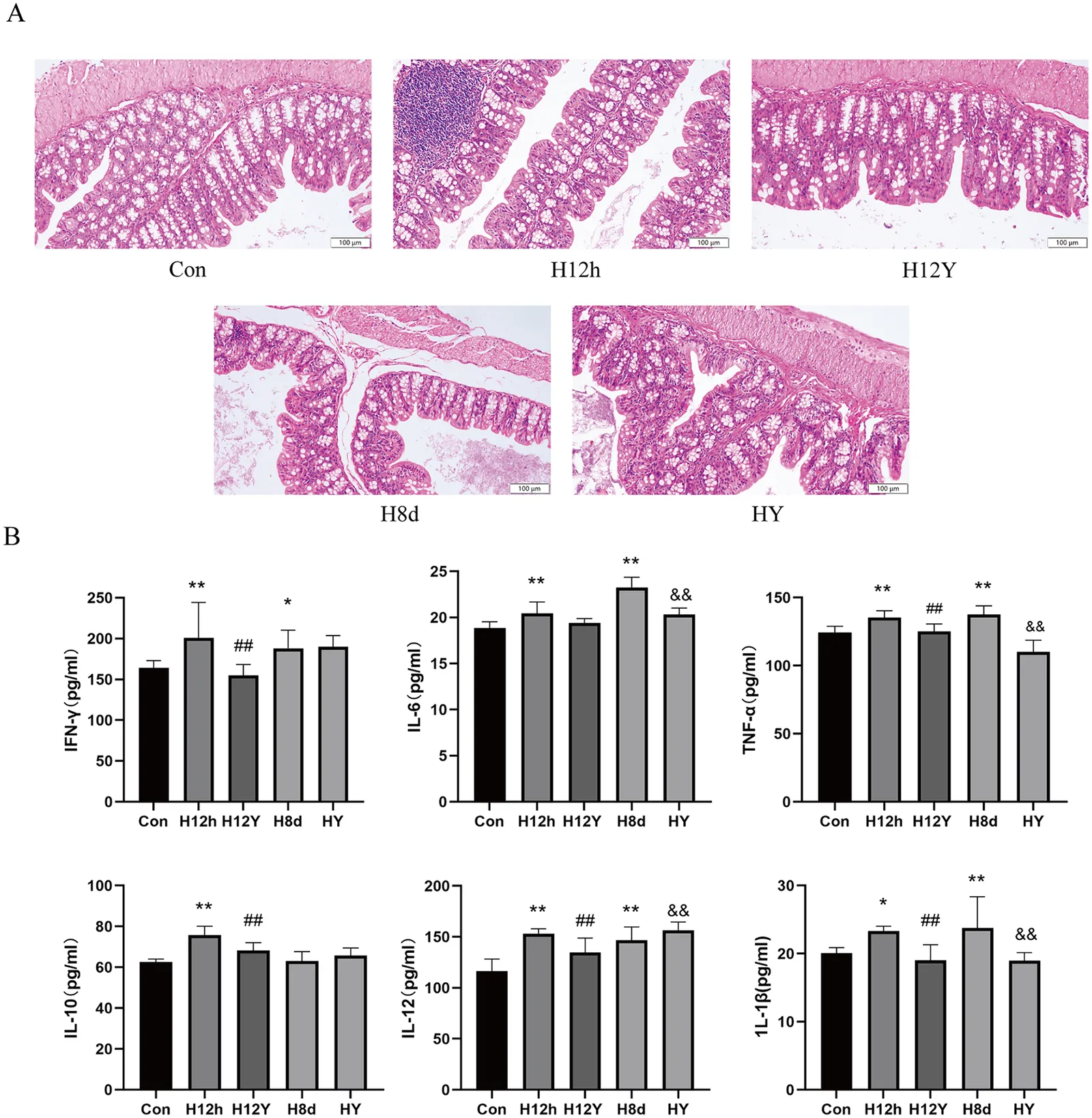
BQT alleviates colonic inflammatory injury induced by high-altitude exposure. **(A)** Histological assessment of colonic mucosa by H&E staining (200×) **(B)** Effect of BQT intervention on colonic inflammatory factors in each experimental group of mice. ^*^Con vs. H12h, H8d, ^*^P < 0.05, ^**^P < 0.01. ^#^H12h vs. H12Y, ^#^*P* < 0.05, ^*##*^*P* < 0.01. ^&^H8d vs. HY, ^&^*P* < 0.05, ^&&^*P* < 0.01.

Hypoxia exposure in high-altitude environments can alter the composition and abundance of gut microbiota, with a significant reduction in the abundance of Firmicutes. Accordingly, BQT containing two Firmicutes strains was selected for intervention in this study. HE staining showed that colonic epithelial cells in the control group were arranged regularly, and mucosal height and mucosal epithelium thickness were at normal levels. Mice in the 12 h high-altitude exposure group exhibited disorganized colonic epithelial cells accompanied by lymphocyte infiltration; in contrast, lymphocyte infiltration was markedly alleviated, and mucosal height and mucosal epithelium thickness tended to be normal in the high-altitude 12 h BQT intervention (H12Y) group. In the high-altitude 8d (H8d) group, obvious lymphocyte infiltration, disorganized colonic epithelial cells, and significant impairment in the morphology and number of goblet cells were observed. By comparison, colonic epithelial cell morphology was significantly improved in the high-altitude BQT (HY) group (Figure 4A). These results suggest that BQT can significantly ameliorate high-altitude induced colonic tissue barrier damage in mice. As shown in Figure 4B, the levels of inflammatory factors in colonic tissues were significantly upregulated in both the 12 h and 8 d high-altitude groups compared with the control group (*P* < 0.05), indicating that high-altitude exposure can induce inflammatory responses in mouse colonic tissues. After intervention with BQT, the levels of inflammatory factors in colonic tissues were downregulated to varying degrees in both the 12 h and 8 d high-altitude BQT groups, with statistically significant differences compared with the corresponding high-altitude model groups (*P* < 0.05). In conclusion, supplementation with BQT can improve the colonic histomorphology and thereby alleviate inflammatory injury in mouse colonic tissues.

### 3.5 Supplementation with BQT improves the protein and mRNA levels of colonic tight junctions under high-altitude exposure

**Figure 5:**
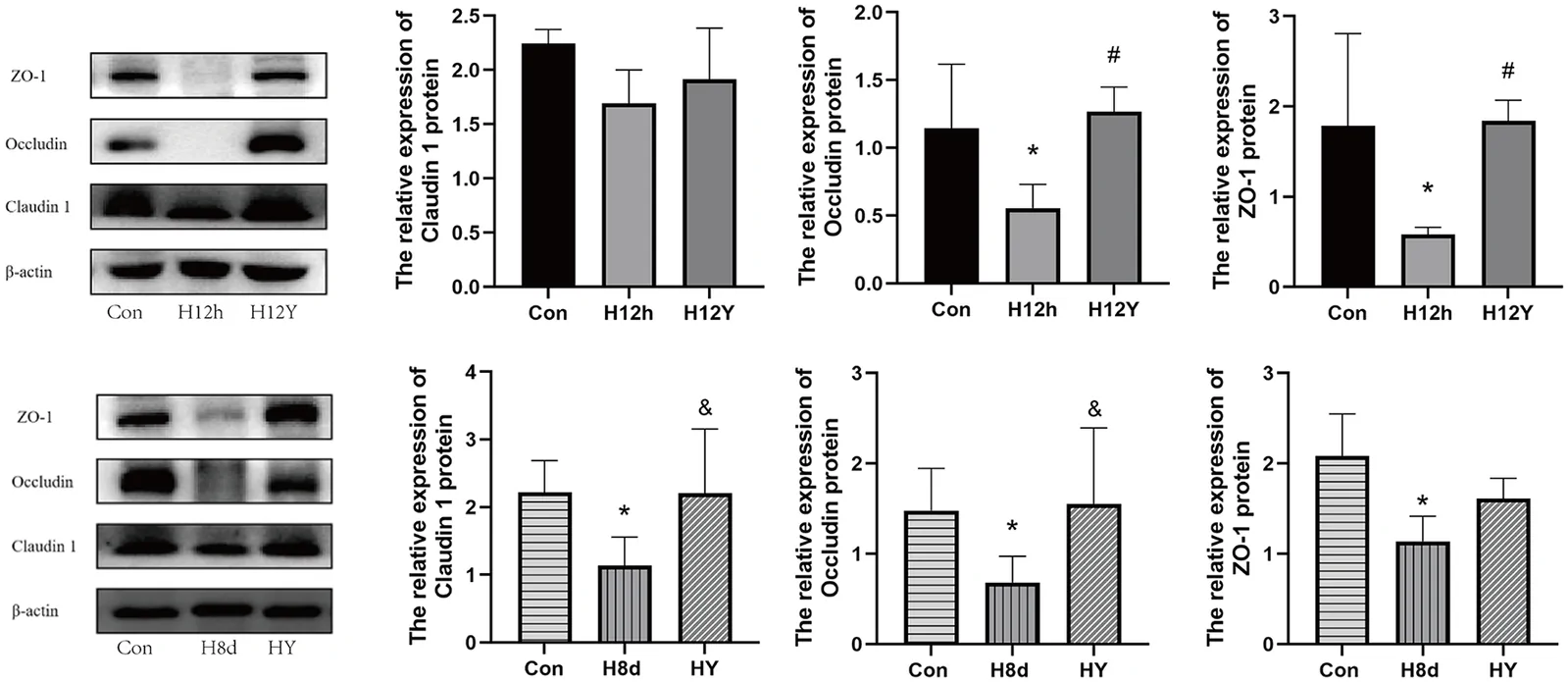
Effects of BQT on the expression of tight junction proteins. **(A–D)** Protein expression levels of tight junction proteins (Claudin-1, Occludin, and ZO-1) after 12 h of BQT treatment. **(E–H)** Protein expression levels after 8 days of BQT treatment. ^*^Con vs. H12h, H8d, ^*^P < 0.05, ^**^P < 0.01. ^#^H12h vs. H12Y, ^#^*P* < 0.05, ^*##*^*P* < 0.01. ^&^H8d vs. HY, ^&^*P* < 0.05, ^&&^*P* < 0.01.

As presented in Figures 5A–D, the expression of colonic tight junction proteins was significantly decreased in high-altitude 12 h group. Compared with the control group, the levels of Claudin-1, Occludin, and ZO-1 were reduced by 24.52, 51.53, and 67.36%, respectively. Following BQT intervention, the expression of these three proteins in high-altitude 12 h BQT group was markedly upregulated by 12.85, 128.51, and 215.49%, respectively. Similarly, as illustrated in Figures 5E–H, Claudin-1, Occludin, and ZO-1 levels were decreased by 48.76, 53.66, and 45.29% in high-altitude 8 d group, suggesting that both acute exposure and adaptive phases of high-altitude stress contribute to colonic barrier disruption in mice. Compared with the high-altitude 8 d group, the BQT group showed significant elevations in Claudin-1, Occludin, and ZO-1 expression by 94.23, 127.04, and 41.43%, respectively. Collectively, these findings demonstrate that administration of BQT effectively attenuates high-altitude induced colonic barrier injury in mice. High-altitude exposure suppresses the expression of colonic tight junction proteins, whereas probiotic supplementation reverses this inhibitory effect and consequently enhances intestinal barrier function.

## 4 Discussion

The host and gut microbiota coexist in a dynamic, mutually beneficial relationship. The host provides a favorable habitat for the microorganisms, which in turn initiate and maintain the hypoxic environment within the intestine, a state crucial for enterocyte nutrient absorption, intestinal barrier function, and immune responses (Kaur et al., 2022; Konjar et al., 2021). However, this mutualistic symbiosis can be disrupted by external environment that alter the gastrointestinal environment and the gut microbiota. Previous studies have shown that high-altitude hypoxia can lead to gut microbiota dysbiosis and impaired intestinal barrier integrity, thereby increasing the risk of bacterial and toxin invasion into the body *via* the intestine (Karl et al., 2025; Liu et al., 2026; McKenna et al., 2022). Nevertheless, the interactive relationship between intestinal barrier injury and gut microbial dysbiosis in the plateau environment has not been fully elucidated. Currently, there are few effective microbiota interventions for intestinal damage caused by high-altitude environments, and the application potential of the clinically approved BQT under hypoxic plateau conditions remains to be explored. In this study, we established a mouse model of high-altitude exposure. Using FMT and BQT intervention, it systematically investigated the central role of the gut microbiota in high-altitude induced colonic inflammation and damage, as well as the targeted regulatory effects of BQT. This provides experimental evidence and novel insights for nutritional microecological intervention in plateau intestinal injury. Gut microbiota diversity is a crucial indicator reflecting intestinal microecological homeostasis, and its structural imbalance is closely associated with the pathogenesis and progression of various intestinal diseases (Corral López et al., 2026). This study found that high-altitude exposure (acute 12-h phase and 8-d adaptation phase) significantly reduced the gut microbiota diversity in mice, accompanied by marked changes in microbial composition. Specifically, this was characterized by a decreased Firmicutes/Bacteroidota (F/B) ratio and an increased abundance of Proteobacteria and Verrucomicrobia, which is largely consistent with findings from previous studies on high-altitude hypoxia. Firmicutes and Bacteroidota, as the two most abundant phyla in the gut microbiota, have a ratio change often closely linked to intestinal metabolism and barrier function. A decrease in the F/B ratio typically suggests dysfunction in the metabolic capacity of the gut microbiota, potentially leading to reduced secretion of beneficial metabolites like short-chain fatty acids (SCFAs; Sankar et al., 2024). SCFAs, the primary metabolic end products of gut microbial fermentation, serve as an essential energy source for intestinal epithelial cells, upregulate the expression of tight junction proteins to fortify intestinal barrier integrity, and suppress the release of pro-inflammatory cytokines, thereby attenuating intestinal inflammation (Donohoe et al., 2011; Morrison and Preston, 2016). Although we did not directly measure SCFAs or other microbial metabolites in this study, the observed amelioration of colonic injury and restoration of barrier function following microbiota modulation raises the possibility that such metabolites may contribute to the protective effects—a hypothesis that warrants further metabolomic investigation. Conversely, an increased abundance of Proteobacteria is considered a significant marker of gut microbiota dysbiosis. Many bacterial strains within this phylum possess pro-inflammatory properties and can induce intestinal inflammatory injury by activating inflammatory signaling pathways in the intestinal mucosa (Lo Sasso et al., 2021; Mukhopadhya et al., 2012). This also explains the exacerbated colonic inflammation observed in mice after high-altitude exposure in this study. Notably, the abundance of Verrucomicrobiota, primarily *Akkermansia*, was significantly increased following high-altitude exposure. While this genus has been reported to play context-dependent roles in intestinal barrier function, the functional relevance of its abundance change in the present study remains unclear and requires further investigation (Tingler and Engevik, 2025).

To clarify the core driving role of gut microbiota in high-altitude induced colonic injury, we established PGF mouse models and FMT models. The results showed that FMT effectively ameliorated the abnormal colonic morphology induced by high-altitude exposure, increased mucosal thickness, decreased levels of intestinal inflammatory factors, and enhanced intestinal barrier function. Colonic mucosal epithelium are critical structures for nutrient absorption and defense against external stimuli, and damage to their morphological integrity results in decreased absorptive function accompanied by increased intestinal mucosal permeability. Excessive release of inflammatory cytokines further exacerbates intestinal mucosal inflammation. By detecting the expression of tight junction proteins and their mRNA transcript levels, we found that the mechanical barrier of the colonic epithelium was significantly strengthened after FMT. This further demonstrates that gut microbiota dysbiosis is a significant trigger for colonic barrier inflammatory injury caused by the high-altitude environment.

FMT, by supplementing with normal gut microbiota, can reconstitute intestinal microecological homeostasis, promote the colonization and proliferation of beneficial bacteria, inhibit the growth of harmful bacteria, thereby reducing the release of pro-inflammatory cytokines, repairing intestinal mucosal epithelium morphology, and enhancing intestinal barrier function. It should be noted that this study did not include a reverse FMT experiment, that is, transplantation of microbiota from high-altitude model mice into healthy naïve recipients. Therefore, we were unable to directly test whether the dysbiotic microbiota induced by high-altitude hypoxia are sufficient to independently transfer the inflammatory phenotype. This limits, to some extent, the causal inference regarding the pathogenic role of the gut microbiota. Future studies incorporating a bidirectional FMT design are warranted to more comprehensively validate the causal relationship between gut dysbiosis and high-altitude colonic injury. Furthermore, although this study demonstrated the overall protective effects of the microbiota through PGF and FMT experiments, we did not identify the specific key functional bacterial taxa or effector metabolites responsible for these effects. The molecular mechanisms underlying microbiota-mediated protection remain unclear and warrant further investigation.

Intestinal barrier dysfunction is a key characteristic of high altitude induced colonic inflammatory injury, and tight junction proteins, as critical structures for inter-cellular connections between intestinal epithelial cells, have their expression levels and mRNA transcription directly determining intestinal mucosal permeability (Saha et al., 2025; Yang et al., 2024; Zhao et al., 2021). The results of this study demonstrate that high-altitude exposure significantly downregulated the protein expression and mRNA transcription levels of ZO-1, Occludin, and Claudin-1 in mouse colonic tissue, confirming the destructive effect of high-altitude hypoxia on intestinal barrier function. However, intervention with BQT effectively reversed the hypoxia induced downregulation of tight junction protein expression and mRNA transcription, and significantly alleviated colonic inflammation in high-altitude exposed mice. This indicates that this probiotic preparation exerts a protective effect against high-altitude colonic injury by repairing the intestinal barrier function, also highlighting its immense potential as an already marketed drug for the prevention and treatment of high-altitude intestinal injury. BQT, a commonly used microecological preparation approved for clinical use, primarily comprises *Bifidobacterium infantis, Lactobacillus acidophilus, Enterococcus faecalis*, and *Bacillus cereus*. The first three are normal gut microbiota constituents in healthy humans; their supplementation allows direct colonization on the intestinal mucosal surface, competitively inhibiting the adhesion and proliferation of pathogenic bacteria. *Bacillus cereus*, upon colonization in the intestine, can consume oxygen, creating a suitable anaerobic environment for anaerobes like *Bifidobacterium infantis* and *Lactobacillus acidophilus*, thereby promoting the colonization and proliferation of beneficial flora and further optimizing the gut microbiota structure (Wang et al., 2025). Compared to other unmarketed probiotic preparations, BQT possess advantages such as mature clinical application and high safety. This study confirms its ability to effectively regulate gut microbiota homeostasis after high-altitude exposure, repair intestinal barrier function, and alleviate intestinal inflammatory responses. This provides a solid experimental basis for extending its use to clinical interventions for high-altitude intestinal injury and suggests that this drug holds promise as a microecological intervention for intestinal barrier damage caused by gut microbiota dysbiosis at high altitude. Furthermore, BQT can regulate intestinal mucosal immunity, inhibiting the release of pro-inflammatory cytokines and promoting the expression of anti-inflammatory cytokines, thereby reducing intestinal inflammation, further clarifying the mechanism by BQT ameliorate highaltitude induced colonic injury.

In conclusion, the present study establishes gut microbiota dysbiosis as a critical contributor to highaltitude-induced colonic inflammatory injury, and provides evidence that the clinically approved BQT exerts protective effects against intestinal injury in mice through restoration of microbial homeostasis and preservation of intestinal barrier integrity. This research not only enriches the understanding of the pathogenesis of plateau intestinal injury but also highlights the significant application potential of BQT, as an already marketed drug, in treating intestinal barrier damage resulting from gut microbiota dysbiosis at high altitudes. It proposes a safe, translatable, and easily promotable nutritional microecological intervention strategy, offering new insights and experimental evidence for the prevention and treatment of intestinal injury in plateau populations. In the future, we will further investigate the specific molecular mechanisms by which gut microbial metabolites mediate plateau-induced colonic injury, aiming to provide support for developing more targeted strategies for intestinal protection at high altitude.

## Data Availability

The data presented in the study are deposited in the NCBI Sequence Read Archive (SRA) repository, accession number PRJNA1519109.
